# Fisher: a program for the detection of H/ACA snoRNAs using MFE secondary structure prediction and comparative genomics – assessment and update

**DOI:** 10.1186/1756-0500-1-49

**Published:** 2008-07-21

**Authors:** Eva Freyhult, Sverker Edvardsson, Ivica Tamas, Vincent Moulton, Anthony M Poole

**Affiliations:** 1 The Linnaeus Centre for Bioinformatics, Uppsala University, Box 598, S-751 24 Uppsala, Sweden; 2 Department of Natural Sciences, Mid Sweden University, S-851 70, Sundsvall, Sweden; 3 Department of Molecular Biology & Functional Genomics, Arrhenius Laboratories for Natural Sciences, Stockholm University, SE-106 91 Stockholm, Sweden; 4 School of Computing Sciences, University of East Anglia, Norwich, NR4 7TJ, UK; 5 School of Biological Sciences, University of Canterbury, Private Bag 4800, Christchurch, New Zealand; 6 Department of Clinical Microbiology, Clinical Bacteriology, Umeå University, 901 85 Umeå, Sweden; 7 Department of Biochemistry & Molecular Biology, Dalhousie University, Halifax, NS, Canada

## Abstract

**Background:**

The H/ACA family of small nucleolar RNAs (snoRNAs) plays a central role in guiding the pseudouridylation of ribosomal RNA (rRNA). In an effort to systematically identify the complete set of rRNA-modifying H/ACA snoRNAs from the genome sequence of the budding yeast, *Saccharomyces cerevisiae*, we developed a program – Fisher – and previously presented several candidate snoRNAs based on our analysis [1].

**Findings:**

In this report, we provide a brief update of this work, which was aborted after the publication of experimentally-identified snoRNAs [2] identical to candidates we had identified bioinformatically using Fisher. Our motivation for revisiting this work is to report on the status of the candidate snoRNAs described in [1], and secondly, to report that a modified version of Fisher together with the available multiple yeast genome sequences was able to correctly identify several H/ACA snoRNAs for modification sites not identified by the snoGPS program [3]. While we are no longer developing Fisher, we briefly consider the merits of the Fisher algorithm relative to snoGPS, which may be of use for workers considering pursuing a similar search strategy for the identification of small RNAs. The modified source code for Fisher is made available as supplementary material.

**Conclusion:**

Our results confirm the validity of using minimum free energy (MFE) secondary structure prediction to guide comparative genomic screening for RNA families with few sequence constraints.

## Findings

Small nucleolar RNAs (snoRNAs) guide nucleotide modifications of ribosomal RNAs (rRNAs), as well as an expanding repertoire of cellular RNAs [4,5]. SnoRNAs can be divided into two broad families: the C/D-box snoRNAs that guide site-specific 2'-O-methylation of ribose [6,7], and the H/ACA snoRNAs [8,9] that guide specific conversions of uridine (U) to pseudouridine (ψ).

Both H/ACA and C/D-box snoRNAs target the exact nucleotide for modification via base-complementarity to their target molecule, thereby permitting sequence-specific binding to the region of the target to be modified. Based on the guide regions as well as other known sequence motifs and structural elements, search tools have been developed both to identify C/D-box [10,11] and H/ACA snoRNAs [1,3,11], and, more recently, snoRNAs with no known target sequence [12,11]. Tools are also available for screening archaeal genomes for snoRNA-like sRNAs [13-15].

**Figure 1:**
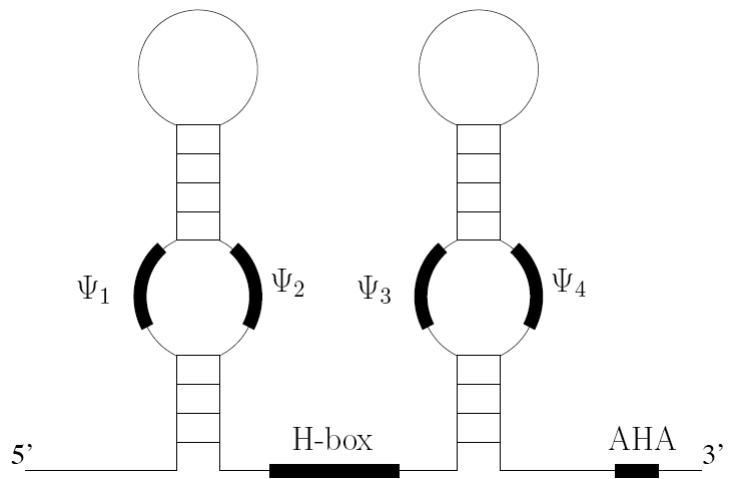
**Idealised secondary structural representation of an H/ACA family snoRNA**. H/ACA snoRNAs generally form a double hairpin structure. Each hairpin carries an internal bulge, called a pseudouridylation pocket, which may possess regions of base complementarity to a target RNA (not all snoRNAs carry two functional pseudouridylation pockets). We have designated these regions ψ_1–4 _for descriptive purposes. Other key motifs are the H-box, located in the hinge region between the two hairpins, and the ACA box at the 3' end of the molecule.

We previously developed the program Fisher as a tool to screen for H/ACA snoRNAs in the genome of the budding yeast, *Saccharomyces cerevisiae *[1]. Since the conserved sequence motifs of the H/ACA box snoRNAs are short (and in the case of regions of intermolecular interaction with rRNA target molecules, discontinuous) (Figure 1) the H/ACA box snoRNAs are harder to identify based on sequence motifs only, and Fisher therefore relies on both primary and secondary structure searches. However, Fisher suffers from a high frequency of false positive predictions (a large number of potential candidates are predicted, but many of these will not be true snoRNAs), and so additional screening of the Fisher candidates is necessary. This is likewise true for the snoGPS program [3].

In this report we describe some minor modifications to Fisher, a brief update on the candidate snoRNAs we reported in Edvardsson et al. [1], and a comparative genome analysis using 14 available yeast genome sequences which heavily reduced the number of false positives and enabled us to identify three yeast snoRNAs not identified by Schattner and colleagues' snoGPS program. We therefore provide a brief comparison of the two search strategies.

The core bioinformatic analyses reported forthwith were completed in 2004, but while we were working to experimentally characterize three candidates arising from our screen, Torchet et al. [2] published a characterization of those same candidates, which they found independently using a lab-based experimental approach. Hence we elected not to pursue the project further. We present the bioinformatics part of our previous study on account of the fact there are ongoing requests for the source code of Fisher, and we therefore wish to provide additional information and results to those originally presented in Edvardsson et al. [1] concerning the potential utility of this program, including confirmation that such a strategy can in principle be used on other datasets. A brief description of the algorithm follows. For a full description, see [1]. For the source code of Fisher, see Additional file 1.

### The *Fisher *algorithm in brief

The essence of the algorithm is as follows. First the search is made for an H-box of the form AN_1_AN_2_N_3_N_4_N_5_. This is then scored using a probabilistic model. If the score is acceptable the H-box is accepted. The algorithm then searches downstream for ψ_3 _and ψ_4 _motifs (see Figure 1, Table S1 in Additional file 2) in appropriate locations. Then the algorithm continues to search for the AHA sequence. The complete H-AHA region together with a variable upstream sequence from the H-box is then passed to the secondary structure filters where acceptable folds are investigated. It is also possible to require a hairpin to the left of the H-box containing ψ_1 _and ψ_2 _motifs (see Figure 1, Table S1 in Additional file 2) in appropriate locations. The candidates are scored depending on both primary and secondary structure.

#### Previously reported candidate snoRNAs

In Edvardsson et al. [1], we presented three possible candidate *S. cerevisiae *snoRNAs, two of which were located in the introns of ribosomal protein genes (coding for RPL43A and RPS11A). A third candidate was overprinted on the coding sequence of the gene coding for snoRNP U3 protein MPP10. Neither these, nor any other of the 50 high-scoring candidates from our initial analysis could be detected using either RT-PCR or Northern Blots, whereas control snoRNAs were readily identified (AMP, P. A. McLenachan, A. R. Gore, A. M. Idicula, unpublished observations). We therefore concluded that the real false positive rate was prohibitively high, despite favourable preliminary results reported in Edvardsson et al. [1].

#### Combining *Fisher *with comparative genome data

**Figure 2:**
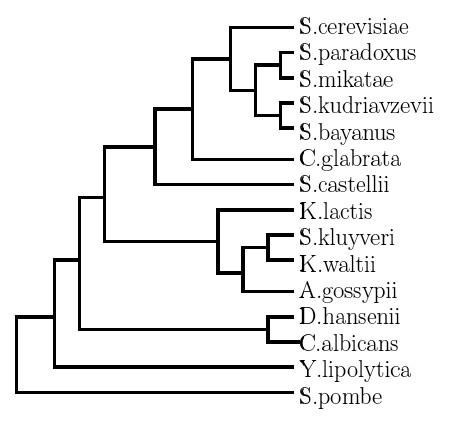
**Evolutionary relationships of the yeast genomes used in the comparative genomics screen**. Schematic based on a tree computed for the 25S rRNA using maximum parsimony, as presented in the supplementary material of Dujon et al. [18] Branch lengths are not preserved from the original maximum parsimony analysis, and only those taxa used in the current analysis are shown.

Next, we made use of genome sequences from 14 additional yeasts, in order to establish whether sequence conservation could be employed as a filter to reduce the high false positive rate. The genomes used were as follows: *S. bayanus, S. mikatae *[16,17], *S. paradoxus, K. waltii *[17], *S. castellii, S. kluyveri, S. kudriavzevii *[16], *C. glabrata, D. hansenii, K. lactis, Y. lipolytica *[18], *C. albicans *[19], *S. pombe *[20] &* A. gossypii *[21] (see Figure 2 for overview of their phylogenetic relationships).

As in Edvardsson et al. [1], the *S. cerevisiae *genome was downloaded from the Saccharomyces Genome Database (SGD) [22], and only a reduced version of the genome was used for the snoRNA screen: i.e. all regions corresponding to open reading frames were removed, with introns added back into the dataset. We modified Fisher to accept ψ4 to ACA distances of between 13 and 16 nucleotides, and allowed any base at the middle position of the ACA-box, which increased the number of potential candidates. We then used blastn [23] to screen the other yeast genomes for sequences homologous to these candidates, using a penalty of nucleotide mismatch of -4, a reward for nucleotide match of +5, and a gap creation and extension penalty of -10. Blast hits shorter than 40 nucleotides, with an identity below 40% or an E-value above 0.01 were not considered as potential homologues. Blast alignments were extended to cover the full length of the S. cerevisiae candidate sequence and realigned using CLUSTAL W [24]. This was done iteratively by adding nucleotides to the homologous sequences and realigning until the alignment covered the full candidate sequence. This gave a list of pairwise alignments between each *S. cerevisiae *candidate and potentially homologous sequences in other yeast genomes, for which pairwise identities could be established.

We compared these results with the results of blasting known *S. cerevisiae *H/ACA snoRNAs against the comparative genome dataset. We found that all snoRNAs known at the time of analysis had homologs (as identified by blastn) in at least three of the four genomes that are most closely related to *S. cerevisiae *(*S. paradoxus, S. mikatae, S. bayanus and S. kudriavzevii*), with high sequence identities (see Table S2 in Additional file 2). The sequence identities were all above 89, 82, 78 and 82%, for *S. paradoxus, S. mikatae, S. bayanus *and *S. kudriavzevii*, respectively. We therefore further examined those candidates where potential homologues were identified in at least three of these four genomes, and with a pairwise sequence identity not below 87, 80, 76 and 80%, respectively.

Sequences in other genomes with high sequence identity to Fisher candidates were examined for the presence of the following features: H-box, ACA-box, and a 3' pseudouridylation pocket (consisting of ψ_3 _and ψ_4 _and elements – See Figure 1, Table S1 in Additional file 2) downstream of the H-box. These features were required to be completely conserved or to at least fulfil the same requirements as specified by Fisher (see [1]). We examined rRNA regions around the pseudouridylated nucleotides in *S. cerevisiae *and confirmed that these were conserved across the yeast species investigated. Hence the ψ_3 _and ψ_4 _and elements making up the pseudouridylation pockets of potentially homologous sequences were all required to be complementary to the same sequence (see Table S1 in Additional file 2). We likewise required the ACA-box to be located at a distance of between 13 and 16 nucleotides from the 5' end of the ψ_4 _element. Finally, we made sure that all homologous sequences had an H-box upstream of the ψ_3 _element at a position corresponding to the H-box position in the aligned candidate sequence, or not more than ten nucleotides upstream or downstream from this position.

We next examined the secondary structure of all candidates that fulfilled the above sequence criteria. H/ACA snoRNAs form a hairpin in the region between H-box and the ACA-box, with the ψ_3 _and ψ_4 _and elements forming an interior bulge (the 3' pseudouridylation pocket). Observation of known snoRNA structures indicated that the region just downstream of ψ_3 _and just upstream of ψ_4 _are always base paired. This observation was investigated in more detail for all known snoRNAs by folding the regions just downstream/upstream of ψ_3 _and ψ_4 _using RNAcofold [25,26]. Table S3 (Additional file 2) shows the number of base pairs predicted for the stem-loop structure formed between the nucleotides immediately 3' and 5' of the ψ_3 _and ψ_4 _elements, and the number of unpaired bases between the pseudouridylation pockets and the stem region immediately above. The investigation showed that every known H/ACA snoRNA with a 3' pseudouridylation pocket have, at a distance of at most one nucleotide from the ψ_3 _and ψ_4 _boxes, a region of at least three complementary bases.

We therefore used these criteria for assessing candidate snoRNA sequences with RNAcofold, and discarded all sequences that did not conform to these folding criteria. Finally a manual check of folding for the complete sequences of all remaining snoRNA candidates served to filter out candidates that did not conform to the expected snoRNA structure.

#### Identification of candidate snoRNAs and conservation across yeast genomes

To be able to compare our results with the results reported by Schattner et al. [3], we performed a search for snoRNAs predicted to guide pseudouridylation at nine sites on *S. cerevisiae *rRNA, for which snoRNAs were identified for six targets (Schattner et al. [3], and three of which remained unidentified at the time of our analyses (maps 10, 13, 15, 22, 32, 35 and 7, 14, 33, respectively – see Table S1 in Additional file 2)). For the six targets studied by Schattner et al., Fisher reported 1590 unique snoRNA candidates. The comparative filters reduced this number to eight candidates (see Table 1). Among these candidates were snR80, snR81 and snR82, i.e. three out of the six novel snoRNAs identified by snoGPS [3]. Two of the novel snoRNAs, snR84 and snR85, cannot be found by Fisher since they only possess a 5' pseudouridylation pocket and Fisher can only identify snoRNAs with a single 3' pocket and snoRNAs carrying two (i.e. both a 5' and a 3') pocket. snR83 possesses a 3' pseudouridylation pocket, but was not among the 1590 candidates. It is rejected by Fisher because it does not fold into a secondary structure with two hairpins. It has a canonical 3' hairpin, but lacks the 5' hairpin and is therefore rejected.

**Table 1:** *S. cerevisiae *candidate snoRNAs found by Fisher for rRNA pseudouridylation sites.

Candidate	Detected on N Blot	ψ-map^a^	ψ-site	Chromosome	Position^b^	Homologues detected in other yeast genomes^c, d^													snoRNA^e^
						Sp	Sm	Sk	Se	Sb	Cg	Sc	Kl	Sl	Kw	Ag	Dh	Ca	

1	N	7	SSU_759_	XV	87507>87772	**+**				**+**									
2	Y	7	SSU_759_	V	52135<52357	**+**	**+**	**+**		**+**	**+**	**+**	**+**		**+**	**+**		**+**	snR80
3	NP	13	SSU_1290_	IV	562489>562750	**+**		**+**											
4	N	14	SSU_1415_	XIII	302531>302787	**+**	**+**												
5	N	14	SSU_1415_	XIII	556582>556828	**+**	**+**	**+**										**+**	
6	NP	15	LSU_776_	II	483358>483595	**+**	**+**	**+**	**+**										
7	Y	15	LSU_776_	V	52135<52357	**+**	**+**	**+**		**+**	**+**	**+**	**+**		**+**	**+**		**+**	snR80
8	NP	22	LSU_1052_	V	517714>517935	**+**	**+**	**+**											
9	NP	22	LSU_1052_	XV	234298>234538		**+**	**+**		**+**									snR81
10	N	33	LSU_2314_	XIV	91706<91951	**+**	**+**	**+**											
11	Y	33	LSU_2314_	XIII	762101<762294	**+**	**+**	**+**		**+**					**+**	**+**			snR86
12	NP	35	LSU_2349_	VII	316840>317063	**+**	**+**	**+**		**+**	**+**	**+**		**+**					snR82
13	NP	35	LSU_2349_	X	519129>519328	**+**	**+**	**+**		**+**									
14	NP	35	LSU_2349_	VIII	271983>272251	**+**		**+**											

^a^corresponds to ψ-map numbering as given in Table S1, Additional file 2.

^b^'>' indicates the candidate is on the Watson strand, and '<' indicates it is on the Crick strand in *S. cerevisiae*.

^c^Abbreviations: Sp – *S. paradoxus*; Sm – *S. mikatae*; Sk – *S. kudriavzevii*; Se – *S. exiguus*; Sb – *S. bayanus*; Cg – *C. glabrata*; Sc – *S. castellii*; Kl – *K. lactis*; Sl – *S. kluyveri*; Kw – *K. waltii*; Ag – *A. gossypii*; Dh – *D. hansenii*; Ca – *C. albicans*.

^d^N.B. no homologues were detected in *Y. lipolytica *or *S. pombe*, though both were screened; *S. exiguus *partial genome sequence from Génolevures [28].

^e^SnoRNAs identified by snoGPS [3] or experimentally [2] which we were also able to identify using Fisher.

^f^Y: detected; N: not detected; NP: Not performed.

For the remaining three target sites (maps 7, 14 and 33 in Table S1, Additional file 2), Fisher reported 2256 candidate snoRNAs for a reduced genome dataset (described in [1]). The comparative filter reduced this number down to two candidates for each target site (Table 1).

**Figure 3:**
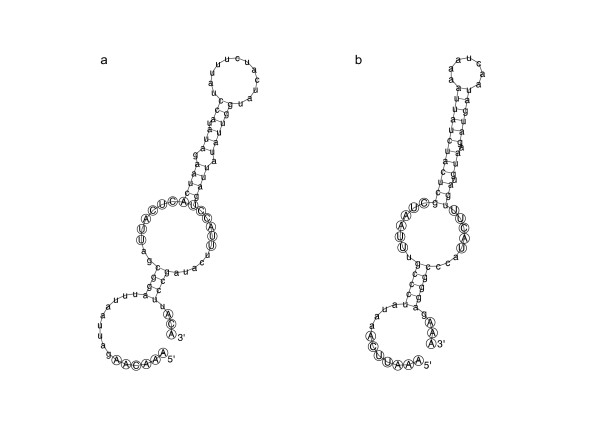
**Secondary structure predictions showing 3' hairpin structures of the two top candidates identified by Fisher**. 3' hairpin structures from H- to ACA-box for **(a) **candidate 7 from Table 1, corresponding to snR80, and **(b) **Candidate 11 from table 1, corresponding to snR86. Residues forming the H-box, ψ-pocket and ACA-box are capitalised and circled.

We subsequently performed Northern blots to probe for expression of these candidates, and found that candidates 2 and 11 were expressed in *S. cerevisiae *(IT & AMP, unpublished observations). Candidate 2 corresponds to snR80, which was determined by Torchet et al. [2] to guide pseudouridylation of SSU_759 _and LSU_776_, and candidate 11 is snR86, which Torchet et al. demonstrated was required for pseudouridylation of LSU_2314_. None of the candidates for position 14 (SSU_1415_) were detected on Northern blots, and we concluded that these are false positives. Torchet et al. demonstrated that this pseudouridylation was guided by snR83, which Fisher fails to detect. As is evident from Table 1, snR80, snR82 and snR86 are conserved in diverse yeast species. Both snR80 and snR86 both form hairpin structures (3' hairpins shown in Figure 3). Alignment of snR80 from *S. cerevisiae *with snR80 sequences from other yeasts, shows that the H-box, ACA-box and the ψ_3 _and ψ_4 _boxes forming the 3' pseudouridylation pocket are almost perfectly conserved, with overall sequence similarity being high among sensu stricto species (*S. cerevisiae, S. paradoxus, S. mikatae, S. kudriavzevii*, and *S. bayanus*) (see Figure S1, Additional file 3). In the case of snR86, only the sequence corresponding to the 3' hairpin is conserved (i.e. from just upstream from the H-box to the 3' end) (see Figure S2, Additional file 3). Based on a comparison between snR86 sequences from *S. cerevisiae *and *C. glabrata*, Torchet et al. proposed that the large 5' region of sn86 was structurally conserved. We examined snR86 sequences from 10 yeast species, and, given the low sequence similarity, uncertainty on the genomic position of the 5'-end for these sequences, and the consequent difficulty in reliably aligning these, we are only confident of the conservation of structure of the 3' region (Figure 3, Figure S2 in Additional file 3).

#### Comparison of our results to those for snoGPS

snoGPS [3] uses a combination of deterministic tests and a probabilistic model to search for snoRNA genes. The program has the option to search for only the 5' stem-loop or only the 3' stem-loop, but as stated in [3] the two-stem alternative is usually used, where a two-hairpin structure is required. snoGPS starts by searching for the guide sequences and the downstream ACA- or H-box. The hairpin structure is investigated by, for example, measuring distances between boxes, and complementarity in the stem regions. For detecting two-stem snoRNAs snoGPS then proceeds to look for a second stem-loop either upstream or downstream of the first. Candidates are then scored using a probabilistic model trained on known snoRNAs. The deterministic tests in snoGPS are generally looser than the corresponding tests in Fisher, but the probabilistic model helps cut down the search space. However, the snoGPS search of the *S. cerevisiae *genome reported in Schattner et al. [3] was complemented with comparative genome analysis and free-energy calculations, to further reduce the number of false candidates. This search resulted in the identification of three new snoRNAs (snR80, snR81, snR85), and confirmation of three additional candidate H/ACA snoRNAs identified previously using QRNA (snR84, snR82, snR83) [27].

The comparative genomics filter presented here significantly reduced the number of snoRNA candidates output by Fisher. As described above, Fisher detected three of the six snoRNAs identified with snoGPS (snR80, snR81, snR82), and snR86, not identified by snoGPS. In addition, Fisher correctly identified snR80 as the candidate responsible for modification at SSU_759_. Given that the deterministic filters used in snoGPS are more generous than the corresponding filters in Fisher, we would expect that the candidates we identify would probably have been excluded due to the probabilistic filters implemented in snoGPS. Another possibility is that the candidates did not pass the comparative genome analysis in [3], which contained a manual evaluation stage.

Comparing the results from our method with the results reported in [3] we conclude that our algorithm is more restrictive, but still at the same level of predictive power. A clear drawback with the Fisher algorithm is that it cannot detect snoRNAs with only a 5' pseudouridylation pocket, providing an obvious explanation as to why we could not identify snR84 and snR85. In the case of snR83, which possesses a 3' pseudouridylation pocket but not a 5' pocket, in silico folding reveals it possesses only a single hairpin structure. As Fisher requires snoRNA candidates to conform to a two-hairpin secondary structure, any snoRNAs with the structural characteristics of snR83 should not be identified. Ironically, snR86 is a very large snoRNA (~1000 nt), and the unusual size and predicted secondary structure reported therein [2] is such that we would expect Fisher to not detect it.

While Fisher reports a double hairpin structure for snR86, a multispecies alignment and secondary structure predictions do not strongly support this. Upstream of the H-box, there is limited conservation across diverse yeast, in contrast to the well-conserved downstream region (see Figure S2 in Additional file 3). It would certainly be possible to modify Fisher to allow single-stem structures. However, a caveat of such a modification would be an increased number of false positives. In that the success of such searches depends heavily upon the availability of multiple genome sequences from related species, an increase in false positive results may not necessarily represent a major obstacle for such datasets.

### Conclusion

Our analyses demonstrate that, in spite of the high false positive rate apparent when screening a single genome, the Fisher algorithm is effective if used in combination with a comparative genomics analysis. The combined approach reported here resulted in a small number of candidates, thereby permitting subsequent experimental screening for bona fide snoRNAs. This report confirms the utility of minimum free energy (MFE) secondary structure prediction as a method for screening for families of structural RNA with few sequence constraints, and demonstrates the effectiveness of combining secondary structure with comparative genome data.

### Availability and requirements

• Project name: Fisher (snoRNA search)

• Project home page: N/A

• Operating system(s): Platform independent

• Programming language: C

• Other requirements:Vienna RNA Package, see

• 

• License: Freeware

• Any restrictions to use by non-academics: None

### Competing interests

The authors declare that they have no competing interests.

### Authors' contributions

EF performed the majority of the computational analyses described herein; AMP performed additional analyses to update results. SE made modifications to Fisher, ran the program, and assisted in analysis of results. IT performed Northern hybridizations for candidates in Table 1. VM & AMP initiated the project, which was developed with input from all authors. EF and AMP analysed results and wrote the manuscript. All authors read and approved the final version.

## Supplementary Material

Additional file 1 The source code of Fisher. Click here for file

Additional file 2 Three target sites, maps 7, 14 and 33. Click here for file

Additional file 3 Figure S1. Click here for file
