# Supplementary material for: Fisher: a program for the detection of H/ACA snoRNAs using MFE secondary structure prediction and comparative genomics – assessment and update
Source: BMC Res Notes. 2008 Jul 21;1:49. doi: 10.1186/1756-0500-1-49 (PMC2551606; doi:10.1186/1756-0500-1-49)
Supplement: Additional file 1 — The source code of Fisher. [file 1756-0500-1-49-S1.zip › fisher_v1.0/psi_map.pdf]

TABLE 1. Regions that are reverse complementary to those flanking the yeast rRNA pseudouridylation sites (see Fig. 1). Column one contains our adopted labeling system of a Ψ-pair. We have identified the locations of rRNA uridines targeted for modification by H/ACA snoRNAs in column 2. The reverse complementary motifs of the rRNA are tabulated in columns three and four (Ψ-pair). Parts of these motifs (including possible wobbles) are expected to be found in snoRNA pseudouridylation pockets. The last column gives predicted or demonstrated Ψ-pairs for the known snoRNAs. Ψ-pair 39 (U_{2826}) might be wrongly assigned to snR34 (W=38), see Results section. Ψ-pair 27 (U_{2133}) is probably also wrongly assigned to snR3, see our Table 4.

Ψ-pair label	Ψ-site	Ψ_1 (Ψ_3)	Ψ_2 (Ψ_4)	snoRNA
18S Subunit				
1	U_{106}	AACGAUAACU	UUUAAUGAGC	snR44
2	U_{120}	GGAACUAUCA	UAAACGAUAA	
3	U_{211}	UUUUUUAUCU	UAAAUACAUC	
4	U_{302}	AUAGGGCAGA	UUUGAAUGAA	
5	U_{466}	UAUUUAUUGU	CUACCUCCCU	snR189
6	U_{632}	CCCAAAGUUC	CUACGAGCUU	
7	U_{759}	UUGAACACUC	AUUUUUUCAA	
8	U_{766}	GCCUGCUUUG	CACUCUAAUU	
9	U_{999}	GACGGUAUCU	UCAUCUUCGA	snR31
10	U_{1181}	GAGUCAAAUU	GCCGCAGGCU	
11	U_{1187}	CGUGUUGAGU	AAUUAAGCCG	snR36
12	U_{1191}	UCCCCGUGUU	GUCAAAUUAA	snR35
13	U_{1290}	AUCACUCCAC	ACUAAGAACG	
14	U_{1415}	GUUAUUGCCU	AACUUCCAUC	
25S Subunit				
15	U_{776}	CUCUUACUCA	UCCAUCCGAA	
16	U_{960}	CUGCUAUCCU	GGGAAACUUC	snR8
17	U_{966}	GAGCUUCUGC	UCCUGAGGGA	
18	U_{986}	UUACCUCAUA	ACUGAUACGA	snR8
19	U_{990}	CGCUUUACCU	UAAAACUGAU	snR49
20	U_{1004}	AACCUCUAAU	UUCGCUUUAC	snR5
21	U_{1042}	UAAAGUUUGA	AUAGGUCAAG	snR33
22	U_{1052}	CUUACAUAUU	AAGUUUGAGA	
23	U_{1056}	ACUUCUUACA	UUUAAAGUUU	snR44
24	U_{1110}	AUGGCCCACU	AAGCUCUUCA	
25	U_{1124}	UCUGCUUACC	AAAUGGCCCA	snR5
26	U_{2129}	AUUAGACAGU	GAUUCCCCUU	snR11
27	U_{2133}	UUUAAUUAGA	GUCAGAUUCC	snR3?
28	U_{2191}	GCACUGGGCA	AAUCACAUUG	snR32
29	U_{2258}	AAGAGAGUCA	GUUACUCCCG	
30	U_{2260}	UUAAGAGAGU	UAGUUACUCC	
31	U_{2264}	UACCUUAAGA	GUCAUAGUUA	snR3
32	U_{2266}	GCUACCUUAA	GAGUCAUAGU	
33	U_{2314}	CUCGUUAAUC	UUCAUGCGCG	
34	U_{2340}	AGAUAGUAGA	GGGACAGUGG	
35	U_{2349}	GGUUUCGCUA	UAGUAGAUAG	
36	U_{2351}	GUGGUUUCGC	GAUAGUAGAU	
37	U_{2416}	AACUAGAGUC	GCUCAACAGG	
38	U_{2735}	UCAUGGUUUG	UUCACACUGA	snR189
39	U_{2826}	UGACUGCCAC	GCCAGUUAUC	snR34?
40	U_{2865}	GACAUCGAAG	UCAAAAAGCA	snR46
41	U_{2880}	AUGAUAGGAA	GCCGACAUCG	snR34
42	U_{2923}	UUAGUGGGUG	CAAUCCAACG	snR10
43	U_{2944}	AAACCCAGCU	CGUUCCCUAU	snR37
44	U_{2975}	AGGGUAAAAC	ACCUGUCUCA	snR42

TABLE 2. The snoRNA training data set. The numbering convention for the demonstrated (d) and predicted (p) Ψ-pair entries are defined in Table 1.

snoRNA	$\Psi_1\Psi_2$	*H box* $A\,N_1\,A\,N_2\,N_3\,N_4\,N_5$	$\Psi_3\Psi_4$	*AHA*
snR3		*AGAUCAA*	*27p, 31d*	*AUA*
snR5	*25d*	*AGACCAA*	*20d*	*ACA*
snR8	*16d*	*AGAGCAA*	*18d*	*AUA*
snR10		*AGAACAA*	*42d*	*ACA*
snR11		*AGAUAAA*	*26p*	*ACA*
snR31		*AGAUUAA*	*9d*	*ACA*
snR32		*AGAUAGA*	*28d*	*ACA*
snR33		*AGAUUGA*	*21d*	*ACA*
snR34	*39p*	*AGAAUAA*	*41d*	*ACA*
snR35	*12p*	*AGAUCAU*		*ACA*
snR36		*AAAACAA*	*11d*	*AUA*
snR42		*AGAUAAA*	*44d*	*ACA*
snR44	*1p*	*AUAUUUA*	*23p*	*AAA*
snR46	*40d*	*AAAUUAA*		*ACA*
snR49	*19p*	*AGAUUAU*		*ACA*
snR189	*5p*	*AGAAUAA*	*38p*	*ACA*

TABLE 3. Frequencies of nucleotides used to score H-boxes (see text).

%	N_1	N_2	N_3	N_4	N_5
A	12.50	25.00	18.75	81.25	87.50
U	6.25	62.50	43.75	6.25	12.50
C	0	6.25	37.50	0	0
G	81.25	6.25	0	12.50	0

TABLE 4. Distances for demonstrated or predicted Ψ-pairs. See Figure 2 for definition of *V*, *W*, *X* and *Y*. Multiple distances for X and Y are possible for snR3, snR5, snR8, snR11 and snR44. Since X+Y is conserved, multiple hits occur for various Ψ_3 motifs.

snoRNA	*V*	*W*	*X*	*Y*	*snoRNA*	*V*	*W*	*X*	*Y*
snR3	-	-	*27*	*48*	*snR5*	*41*	*14*	*40*	*51*
	-	-	*38*	*37*		*41*	*14*	*45*	*46*
snR8	*37*	*15*	*21*	*71*		*41*	*14*	*50*	41
	37	*15*	*33*	*59*		*41*	*14*	*56*	*35*
snR11	-	-	*32*	*43*		*41*	*14*	*69*	*22*
	-	-	*36*	*39*	*snR10*	-	-	*25*	*73*
	-	-	*53*	*22*	*snR31*	-	-	*26*	*70*
snR32	-	-	*24*	*35*	*snR33*	-	-	*32*	*33*
snR34	-	-	*28*	*44*	*snR35*	*29*	*16*	-	-
snR36	-	-	*23*	*40*		*26*	*16*	-	-
snR42	-	-	*61*	*59*	*snR44*	*38*	*14*	*32*	*45*
snR46	*37*	*14*	-	-		*38*	*14*	*47*	*30*
snR49	*32*	*14*	-	-	*snR189*	*33*	*15*	*26*	*60*

TABLE 5. The computed results of a Ψ-pair search for the known snoRNAs in our data set. See Table 1 for the definition of our Ψ-pair entries (1-14 represent the pseudouridylation sites on the small rRNA subunit, 15-44 the large subunit). Underlined entries correspond to pseudouridylation sites in the Fournier data base (Samarski and Fournier, 1999); The letters *p* and *d* stand for *predicted* and *demonstrated* assignments, respectively. Bracketed entries have no corresponding $\Psi_1\Psi_2$-pair.

snoRNA	$\Psi_1\Psi_2$	$\Psi_3\Psi_4$
snR3	*2, 3*	*12, 27p, 31d*
snR5	*25d*	*8, 20d, 33, (38)*
snR8	*16d*	*18d*
snR10	-	*(42d)*
snR11	*26*	*11, (26p), (33)*
snR31	*21*	*1, (9d), 23*
snR32	-	*(28d)*
snR33	*1, 4, 14, 20, 23, 33*	*21d, 30*
snR34	-	*(12), (31), (41d)*
snR35	*12p*	-
snR36	*1, 14, 17, 23, 24*	*11d*
snR42	*3*	*44d*
snR44	*1p*	*23p*
snR46	*40d*	*9, 14, 17*
snR49	*2, 3, 19p*	*4, 23*
snR189	*5p*	*38p*

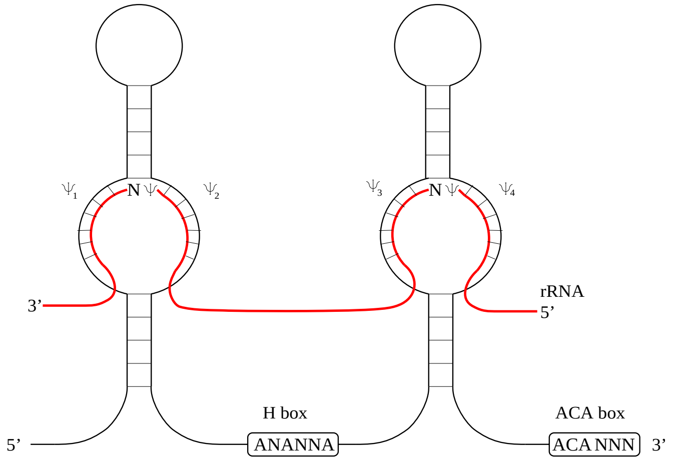

FIGURE 1. An idealistic representation of the consensus primary and secondary structural elements of the H/ACA box snoRNA. Note the classical hairpin-hinge-hairpin-tail secondary structure and the internal loop structures termed the pseudouridylation pockets (Ganot et al. 1997). The corresponding part of the rRNA is also shown. Ψ_i refers to the parts of the snoRNA that are complementary to the rRNA.

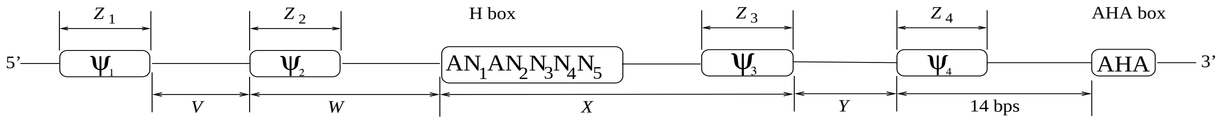

FIGURE 2. Primary structure model used to search for putative snoRNAs consisting of an H-box, an ACA-box (here denoted AHA) and four regions of complementarity to the rRNA subsequences flanking some pseudouridylation site on rRNA (denoted by $\Psi_1\Psi_2$ and $\Psi_3\Psi_4$). Our model requires: $X+Y+14 \leq 142$; $16 \leq X \leq 70$; $Y \geq 30$; $3 \leq Z_3, Z_4 \leq 10$; $Z_3 + Z_4 \geq 9$; $20 \leq V \leq 100$; $11 \leq W \leq 17$; $3 \leq Z_1, Z_2 \leq 10$; $Z_1 + Z_2 \geq 9$.

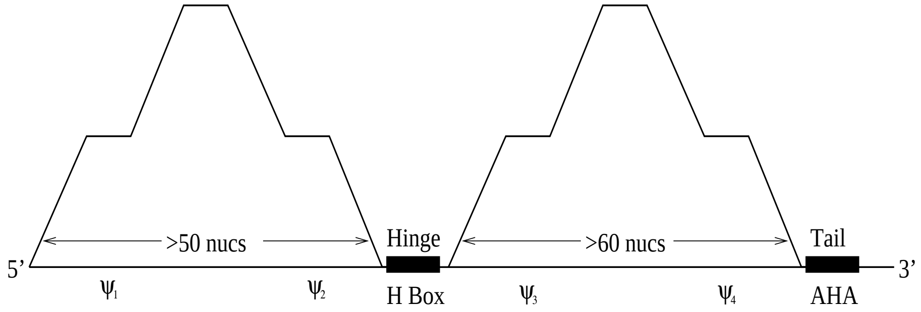

FIGURE 3. Secondary structure model of H/ACA snoRNA. It consists of two ’mountains’ with widths as indicated.

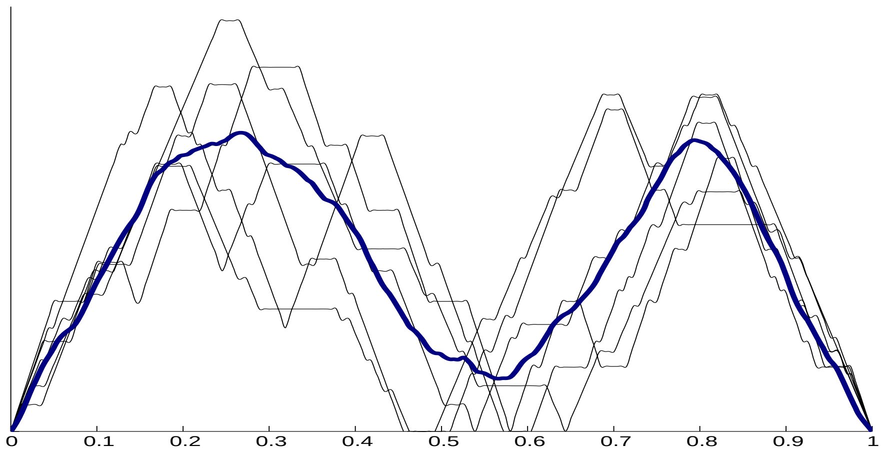

FIGURE 4. Normalised mountain plots (L=100) for 6 of the yeast snoRNAs (snR5, snR10, snR31, snR32, snR33, snR34). To keep the figure clear we only display a subset of the known snoRNAs. The thick line represents the mean structure for the whole snoRNA data-set.

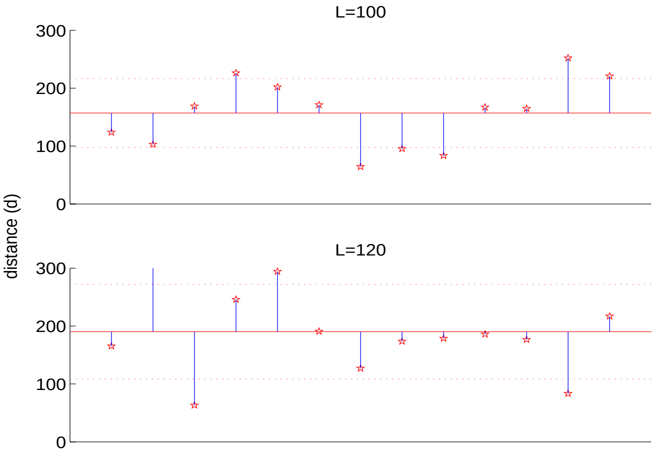

FIGURE 5. snoRNA secondary structure distances (d). The horizontal line is the mean distance $(\overline{d})$. From left to right snoRNA's: 3, 5, 8, 10, 11, 31, 32, 33, 34, 36, 42, 44, 189. The dotted lines represent the standard deviations (±SD).

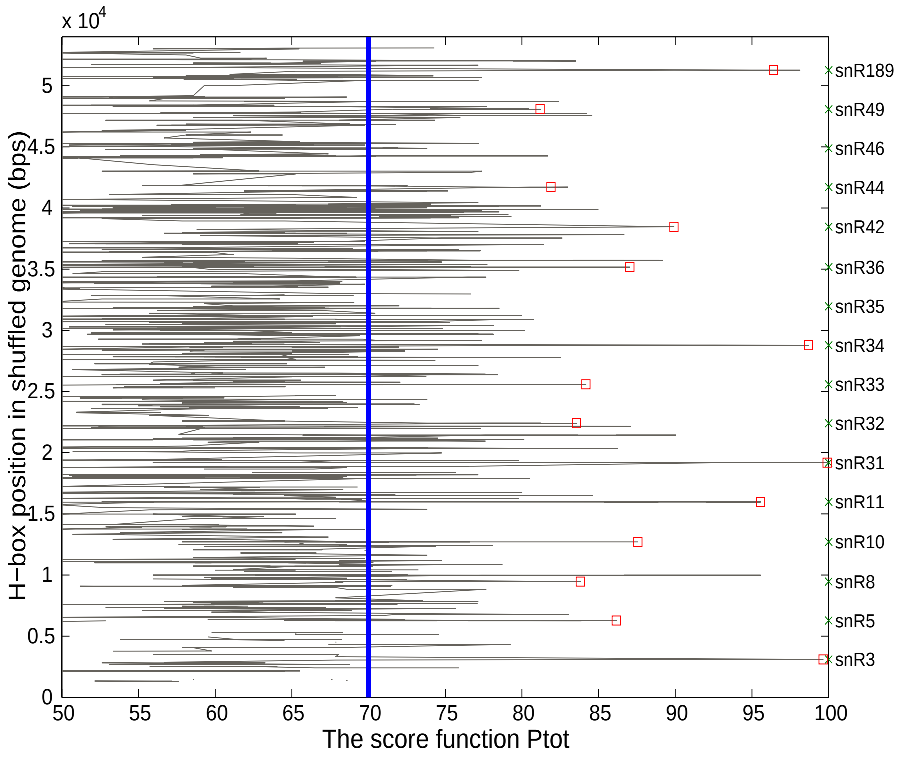

FIGURE 6. The total score (P_{tot}) for the hits in the shuffled test genome. The H-box positions for the 16 snoRNAs are marked with x's. If the hit corresponds to a snoRNA we mark it with a square. For a normal search we would only accept hits scoring above 70 (marked with the bold line).

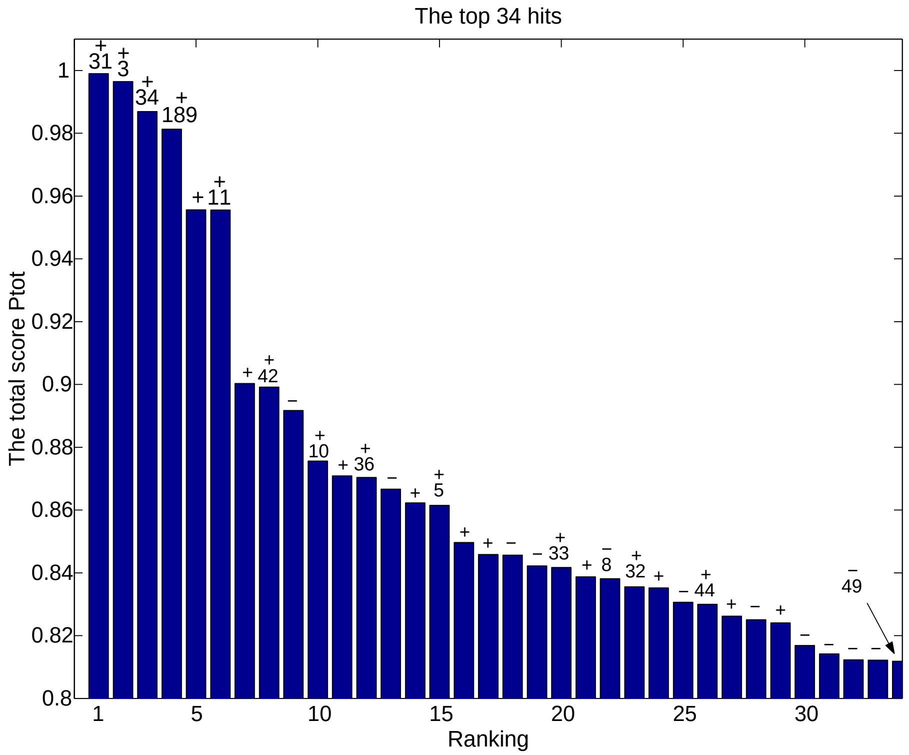

FIGURE 7. The ranking list. The number 31, for example, refers to snR31. The hits that simultaneously had a high primary and secondary score are marked with a ’+’; those that did not are marked with a ’-’.

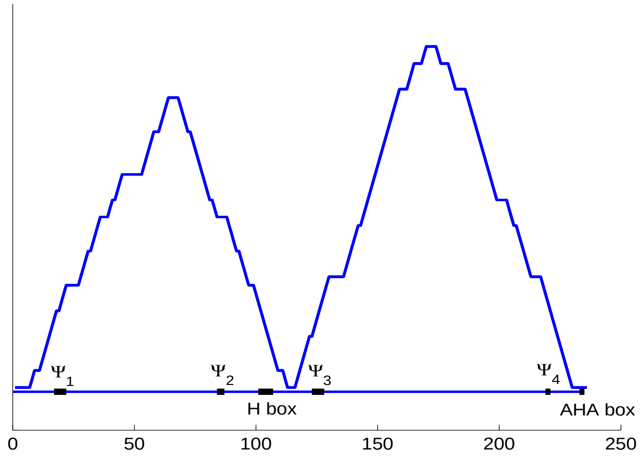

UAUAUUUCUAGUUGUU**CCUUAA**CAAAAUUUUUCAUUCAAUGGCAUAAAUCACUUUACACG
UUCUCGAGAACAGUAGAUGCGAU**GAGU**AUGAUAAAUAGUA**AGAAUAA**UAUGAUAAUGUAC
G**CUAUUA**UAUCGUCAGUACAUAGAUUUUUUGUUUUUAUAAAUGAUGUUCUAUAGAUGUUC
ACAUGAUGAAAGCGGGGAUGAUAUUAAUGUACAAAUUG**UAA**UAUGUGCGAAC**ACA**

FIGURE 8. An example of a typical hit in the yeast genome. This particular hit has both a $\Psi_1\Psi_2$ (32) and a $\Psi_3\Psi_4$-pair (2). In increasing order, the bold motifs in the above sequence are: Ψ_1, Ψ_2, H-box, Ψ_3, Ψ_4 and AHA-box.

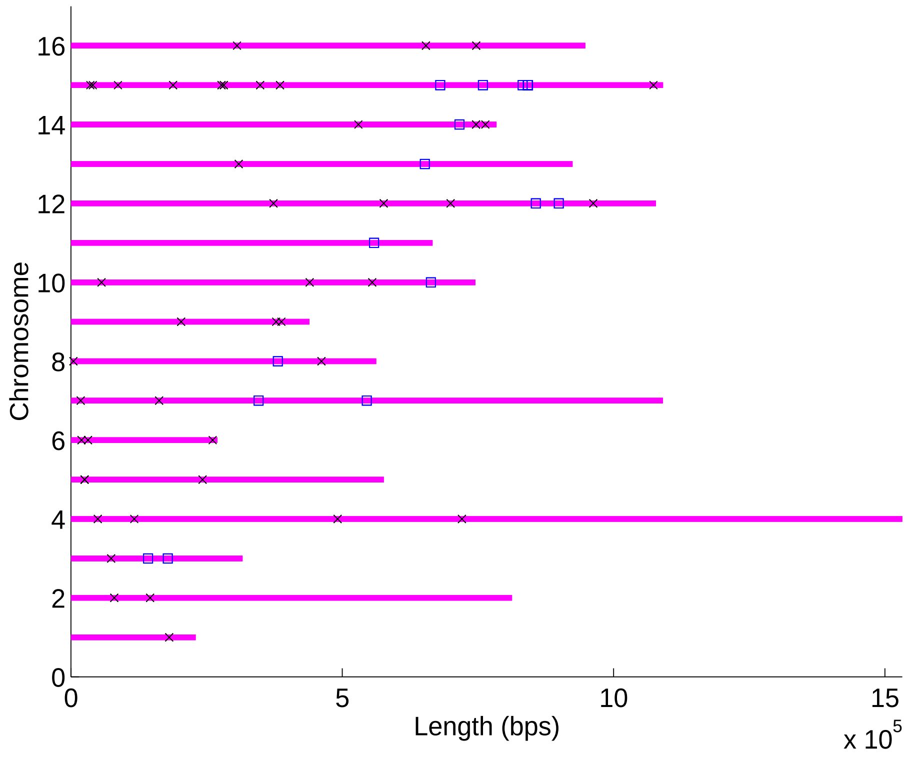

FIGURE 9. snoRNA and putative snoRNA locations in the yeast chromosomes. x-axis: length of chromosome in bases. y-axis: chromosome number. The known snoRNAs are represented by squares and the putative snoRNAs by triangles. The squares indicate known snoRNAs, and the squares indicate putative snoRNAs.
