# Supplementary material for: Fisher: a program for the detection of H/ACA snoRNAs using MFE secondary structure prediction and comparative genomics – assessment and update
Source: BMC Res Notes. 2008 Jul 21;1:49. doi: 10.1186/1756-0500-1-49 (PMC2551606; doi:10.1186/1756-0500-1-49)
Supplement: Additional file 2 — Three target sites, maps 7, 14 and 33. [file 1756-0500-1-49-S2.doc]

**Additional file 2 — Freyhult et al., BMC Research Notes.**

**Table S1: Sequences that are the reverse complement of those flanking pseudouridylation sites in *S. cerevisiae* rRNA^a^**

| ψ-pair label^b^ | ψ-site^c^ | ψ_1_ (ψ_3_)^d^ | ψ_2_ (ψ_4_)^d^ | snoRNA |
| --- | --- | --- | --- | --- |
| 1 | SSU106 | AACGAUAACU | UUUAAUGAGC | snR44 |
| 2 | SSU120 | GGAACUAUCA | UAAACGAUAA | snR49 |
| 3 | SSU211 | UUUUUUAUCU | UAAAUACAUC | snR49 |
| 4 | SSU302 | AUAGGGCAGA | UUUGAAUGAA | snR49 |
| 5 | SSU466 | UAUUUAUUGU | CUACCUCCCU | snR189 |
| 6 | SSU632 | CCCAAAGUUC | CUACGAGCUU | snR161 |
| 7 | SSU759 | UUGAACACUC | AUUUUUUCAA | snR80 |
| 8 | SSU766 | GCCUGCUUUG | CACUCUAAUU | snR161 |
| 9 | SSU999 | GACGGUAUCU | UCAUCUUCGA | snR31 |
| 10 | SSU1181 | GAGUCAAAUU | GCCGCAGGCU | snR85 |
| 11 | SSU1187 | CGUGUUGAGU | AAUUAAGCCG | snR36 |
| 12 | SSU1191 | UCCCCGUGUU | GUCAAAUUAA | snR35 |
| 13 | SSU1290 | AUCACUCCAC | ACUAAGAACG | snR83 |
| 14 | SSU1415 | GUUAUUGCCU | AACUUCCAUC | snR83 |
| 15 | LSU776 | CUCUUACUCA | UCCAUCCGAA | snR80 |
| 16 | LSU960 | CUGCUAUCCU | GGGAAACUUC | snR8 |
| 17 | LSU966 | GAGCUUCUGC | UCCUGAGGGA | snR43 |
| 18 | LSU986 | UUACCUCAUA | ACUGAUACGA | snR8 |
| 19 | LSU990 | CGCUUUACCU | UAAAACUGAU | snR49 |
| 20 | LSU1004 | AACCUCUAAU | UUCGCUUUAC | snR5 |
| 21 | LSU1042 | UAAAGUUUGA | AUAGGUCAAG | snR33 |
| 22 | LSU1052 | CUUACAUAUU | AAGUUUGAGA | snR81 |
| 23 | LSU1056 | ACUUCUUACA | UUUAAAGUUU | snR44 |
| 24 | LSU1110 | AUGGCCCACU | AAGCUCUUCA | snR30 |
| 25 | LSU1124 | UCUGCUUACC | AAAUGGCCCA | snR5 |
| 26 | LSU2129 | AUUAGACAGU | GAUUCCCCUU | snR3 |
| 27 | LSU2133 | UUUAAUUAGA | GUCAGAUUCC | snR3 |
| 28 | LSU2191 | GCACUGGGCA | AAUCACAUUG | snR32 |
| 29 | LSU2258 | AAGAGAGUCA | GUUACUCCCG | snR191 |
| 30 | LSU2260 | UUAAGAGAGU | UAGUUACUCC | snR191 |
| 31 | LSU2264 | UACCUUAAGA | GUCAUAGUUA | snR3 |
| 32 | LSU2266 | GCUACCUUAA | GAGUCAUAGU | snR84 |
| 33 | LSU2314 | CUCGUUAAUC | UUCAUGCGCG | snR86 |
| 34 | LSU2340 | AGAUAGUAGA | GGGACAGUGG | snR9 |
| 35 | LSU2349 | GGUUUCGCUA | UAGUAGAUAG | snR82 |
| 36 | LSU2351 | GUGGUUUCGC | GAUAGUAGAU | snR82 |
| 37 | LSU2416 | AACUAGAGUC | GCUCAACAGG | snR11 |
| 38 | LSU2735 | UCAUGGUUUG | UUCACACUGA | snR189 |
| 39 | LSU2826 | UGACUGCCAC | GCCAGUUAUC | snR34 |
| 40 | LSU2865 | GACAUCGAAG | UCAAAAAGCA | snR46 |
| 41 | LSU2880 | AUGAUAGGAA | GCCGACAUCG | snR34 |
| 42 | LSU2923 | UUAGUGGGUG | CAAUCCAACG | snR10 |
| 43 | LSU2944 | AAACCCAGCU | CGUUCCCUAU | snR37 |
| 44 | LSU2975 | AGGGUAAAAC | ACCUGUCUCA | snR42 |

^a^Updated from the supplementary information in [1] to include results reported in [2].

^b^Numbering scheme to identify pseudouridylation pocket sequences, from [1].

^c^Locations of known pseudouridines in small (SSU) and large subunit (LSU) rRNAs from *S. cerevisiae*.

^d^Reverse complement of the sequences flanking each rRNA pseudouridylation site. Parts of these motifs (which may exhibit mispairing or G-U pairs) are either known or expected to be found in snoRNAs responsible for guiding each modification.

**Table S2: Percent sequence identities between known snoRNAs in *S. cerevisiae*^a^ and their homologues (found by blastn) in the four genomes *S. paradoxus*, *S. bayanus,* *S. mikatae* and *S. kudriavzevii*.**

|  | *S. paradoxus* | *S. bayanus* | *S. mikatae* | *S. kudriavzevii* |
| --- | --- | --- | --- | --- |
| snR3 | 99.0 | 94.9 | 93.3 | 95.9 |
| snR5 | 98.0 | 97.0 | 92.9 | 95.4 |
| snR8 | 97.4 | 94.8 | 92.3 | 95.8 |
| snR10 | 96.7 | 94.3 | 94.7 | 95.9 |
| snR11 | 96.2 | 92.3 | 86.6 | 93.5 |
| snR31 | 92.4 | 91.5 | 88.5 | 85.3 |
| snR32 | 93.2 | 86.8 | 88.0 | 89.0 |
| snR33 | 92.9 | 87.5 | 87.2 | 88.6 |
| snR34 | 96.1 | 94.7 | 94.2 | 93.7 |
| snR35 | — | 89.8 | 84.6 | 87.9 |
| snR36 | 91.2 | 85.2 | 83.5 | 83.0 |
| snR42 | 89.7 | 82.2 | 83.4 | 82.6 |
| snR44 | 92.9 | 91.9 | 87.7 | 89.6 |
| snR46 | 91.1 | 86.6 | 88.4 | 84.4 |
| snR49 | 95.9 | 90.1 | 90.6 | 90.1 |
| snR161 | 94.9 | 87.5 | 78.9 | 82.5 |
| snR189 | 96.4 | 90.6 | 88.6 | 88.9 |
| NOG2 | 92.4 | 88.4 | 89.2 | 92.0 |

^a^This list represents only those snoRNAs used in developing our analysis. For a full list of yeast snoRNAs, please refer to the Yeast snoRNA database [3] at: http://people.biochem.umass.edu/fournierlab/snornadb/

**Table S3: Base pairing potential for the upper stem that forms between nucleotides adjacent to the pseudouridylation pocket for known snoRNAs from *S. cerevisiae*^a^**

| snoRNA | ψ_1,2_-map^b^ | Number of base pairs  (distance to ψ_1_, ψ_2_)^c^ | ψ_3,4_-map^b^ | Number of base pairs  (distance to ψ_3_, ψ_4_)^c^ |
| --- | --- | --- | --- | --- |
| snR3 | 26 | 4 (0,0) | 31,27 | 8 (0,0) |
| snR5 | 25 | 3 (1,1)^d^ | 20 | 5 (0,0) |
| snR8 | 16 | 5 (0,0) | 18 | 3 (0,1) |
| snR9 | 34^e^ |  |  |  |
| snR10 |  |  | 42 | 4 (0,0) |
| snR11 | 37 | 4 (0,0) | 26 | 4 (1,1) |
| snR31 |  |  | 9 | 8 (0,0) |
| snR32 | 28 | 12 (0,0)^d^ |  |  |
| snR33 |  |  | 21 | 9 (0,0) |
| snR34 | 39 | 9 (0,0) | 41 | 4 (0,0) |
| snR35 | 12 | 12(0,0) |  |  |
| snR36 |  |  | 11 | 4 (0,0) |
| snR37 |  |  | 43^e^ |  |
| snR42 |  |  | 44 | 10 (0,0) |
| snR43 | 17 | 10 (0,0) |  |  |
| snR44 | 1 | 10 (0,0) | 23 | 4 (0,0) |
| snR45 | 40 | 11 (0,0) |  |  |
| snR49 | 19,3 | 5 (0,0) | 4 | 11 (0,0) |
| snR49 | 2^e^ |  |  |  |
| snR161 | 8 | 11 (0,0) | 6 | 10 (1,1) |
| snR189 | 5 | 7 (0,0) | 38 | 4 (1,1) |
| snR191 | 29 | 10 (0,0)^d^ | 30 | 5 (0,0)^d^ |
| snR80 |  |  | 15 | 4 (0,0) |
| snR81 |  |  | 22 | 5 (0,0) |
| snR82 |  |  | 35,36 | 3 (1,1) |
| snR83 |  |  | 13 | 8 (0,0) |
| snR84 | 32 | 4 (0,0) |  |  |
| snR85 | 10 | 4 (1,1) |  |  |

^a^For each hairpin, the upper stem consists of bases immediately 3' of motifs ψ_1_ or ψ_3_ and those immediately 5' of motifs ψ_2_ or ψ_4_.

^b^The ψ-map number (from Table S1) corresponding to each pseudouridylation site is given (columns 2 & 4).

^c^Number of predicted base pairs, with the number of unpaired bases between the top of the pseudouridylation pocket and the beginning of the upper stem region in parentheses.

^d^Base pairings were not fully predicted by RNAcofold; some GU base pairs were missing.

^e^due to uncertainties in ψ-box motif positions, the analysis could not be performed.

**References**

1. Edvardsson S, Gardner PP, Poole AM, Hendy MD, Penny D, Moulton V: **A search for H/ACA snoRNAs in yeast using MFE secondary structure prediction.** *Bioinformatics* 2003, **19:**865-873.

2. Schattner P, Decatur WA, Davis CA, Ares M, Jr., Fournier MJ, Lowe TM: **Genome-wide searching for pseudouridylation guide snoRNAs: analysis of the Saccharomyces cerevisiae genome.** *Nucleic Acids Res* 2004, **32:**4281-4296.

3. Piekna-Przybylska D, Decatur WA, Fournier MJ: **New bioinformatic tools for analysis of nucleotide modifications in eukaryotic rRNA.** *RNA* 2007, **13:**305-312.
