# Supplementary material for: Fisher: a program for the detection of H/ACA snoRNAs using MFE secondary structure prediction and comparative genomics – assessment and update
Source: BMC Res Notes. 2008 Jul 21;1:49. doi: 10.1186/1756-0500-1-49 (PMC2551606; doi:10.1186/1756-0500-1-49)
Supplement: Additional file 3 — Figure S1. [file 1756-0500-1-49-S3.pdf]

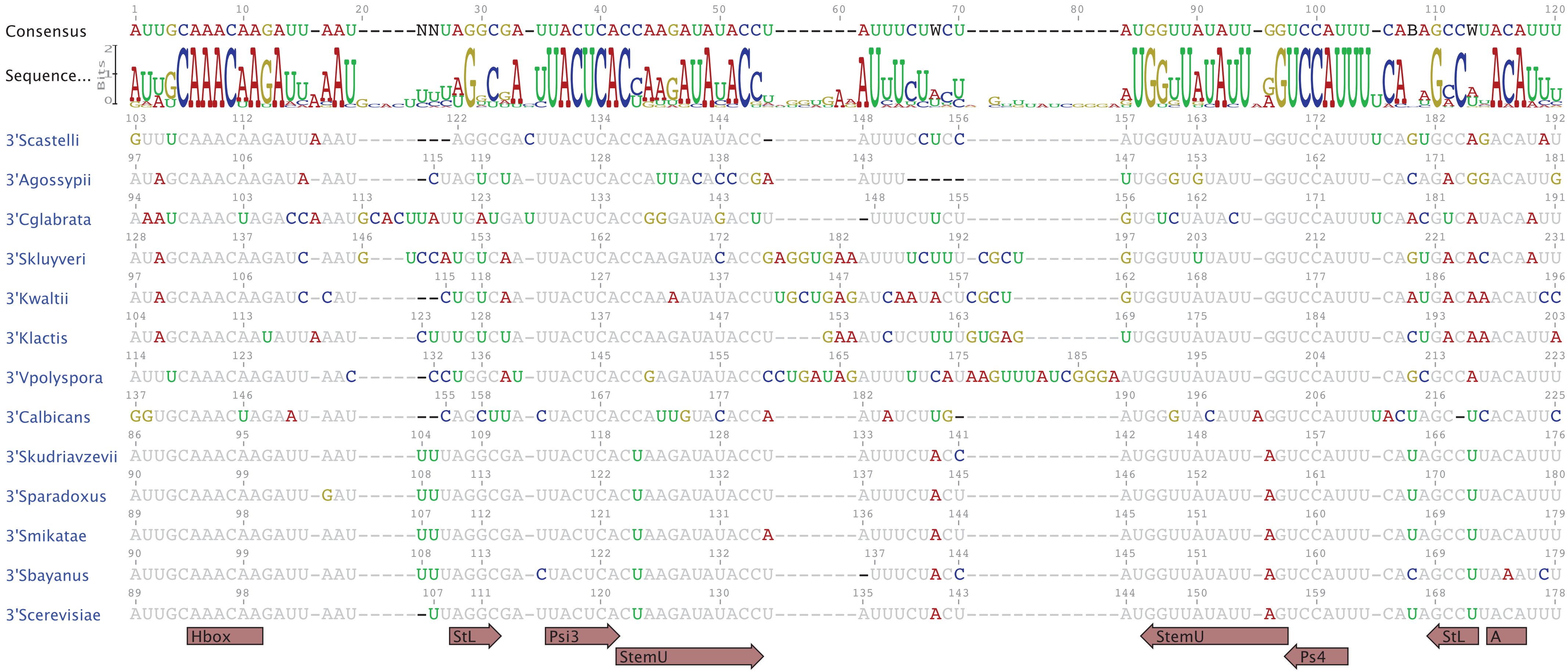

Figure S1: Multiple sequence alignment of the 3' half of snR80.

Conservation of snR80 across diverse yeast, showing conserved sequence and structural features. Sequence differences are highlighted. Most represent compensatory changes that preserve structure and none obviously disrupt functional features. We therefore predict all sequences are functional. The dataset was expanded from that in Table 1, to include additional snR80 candidate sequences, identified using blastn. Alignment was generated using Muscle (Edgar RC: MUSCLE: multiple sequence alignment with high accuracy and high throughput. Nucleic Acids Res 2004, 32:1792-1797), then manually edited. Key structural motifs (brown) are: H-box (Hbox), lower (StL) and upper (StemU) stem regions, 3' pseudouridylation pocket (Psi3, Psi4), ACA box (A). Motifs were derived from sequence data, and secondary structure prediction for the S. cerevisiae sequence computed with RNAfold. Strict (50%) consensus sequence and sequence logo generated using Geneious 3.6 (www.geneious.com) and are shown above the alignment. Abbreviations (+ genbank accession): Scastelli — *Saccharomyces castelli* (AACF01000010); Agossypii — *Ashbya gossypii* (NC_005782); Cglabrata — *Candida glabrata* (NC_006029); Skluyveri — *Saccharomyces kluyveri* (AACE03000001); Kwaltii — *Kluyveromyces waltii* (AADM01000161); Klactis — *Kluyveromyces lactis* (NC_006037); Vpolyspora — *Vanderwaltozyma polyspora* (NZ_AAZN01000148); Calbicans — *Candida albicans* (WGS:AAFO); Skudriavzevii — *Saccharomyces kudriavzevii* (AACI02000833); Sparadoxus — *Saccharomyces paradoxus* (AABY01000108); Smikatae — *Saccharomyces mikatae* (AABZ01000047); Sbayanus — *Saccharomyces bayanus* (AACG02000007); Scerevisiae — *Saccharomyces cerevisiae* (U18795).

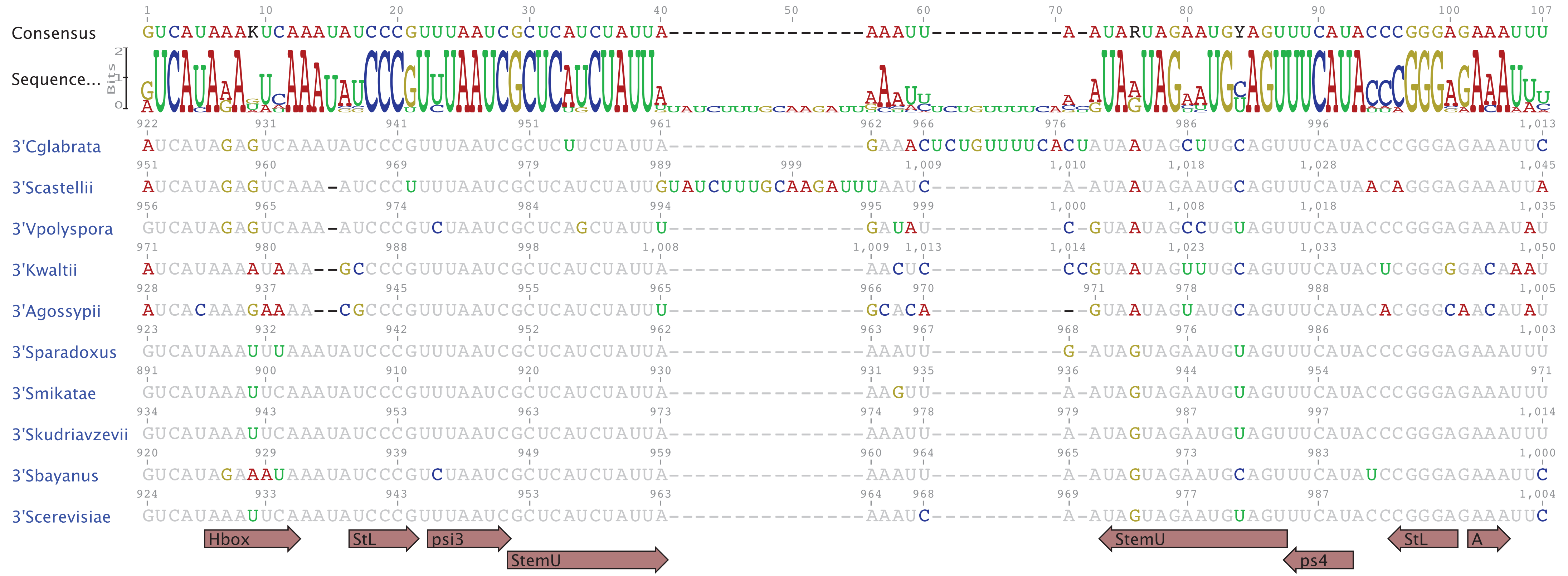

Figure S2: Multiple sequence alignment of the 3' region of snR86.
For description of features and alignment generation, see legend to figure S1. Abbreviations (+ genbank accession): Cglabrata — *Candida glabrata* (NC_006034); Scastelli — *Saccharomyces castelli* (AACF01000020); Vpolyspora — *Vanderwaltozyma polyspora* (NZ_AAZN01000059); Kwaltii — *Kluyveromyces waltii* (AADM01000068); Agossypii — *Ashbya gossypii* (NC_005783); Sparadoxus — *Saccharomyces paradoxus* (AABY01000081); Smikatae — *Saccharomyces mikatae* (AABZ01000413); Skudriavzevii — *Saccharomyces kudriavzevii* (AACI02000062); Sbayanus — *Saccharomyces bayanus* (AACA01000054); Scerevisiae — *Saccharomyces cerevisiae* (Z48756).
